# Amoeba-inspired analog electronic computing system integrating resistance crossbar for solving the travelling salesman problem

**DOI:** 10.1038/s41598-020-77617-7

**Published:** 2020-11-27

**Authors:** Kenta Saito, Masashi Aono, Seiya Kasai

**Affiliations:** 1 Research Center for Integrated Quantum Electronics and Graduate School of Information Science and Technology, Hokkaido University, Sapporo, Japan; 2 Amoeba Energy Co., Ltd., Fujisawa, Japan; 3 Graduate School of Media and Governance, Keio University, Fujisawa, Japan; 4 Graduate School of Science and Technology, Keio University, Yokohama, Japan; 5 Center for Human Nature, Artificial Intelligence, and Neuroscience, Hokkaido University, Sapporo, Japan

**Keywords:** Electrical and electronic engineering, Electronics, photonics and device physics

## Abstract

Combinatorial optimization to search for the best solution across a vast number of legal candidates requires the development of a domain-specific computing architecture that can exploit the computational power of physical processes, as conventional general-purpose computers are not powerful enough. Recently, Ising machines that execute quantum annealing or related mechanisms for rapid search have attracted attention. These machines, however, are hard to map application problems into their architecture, and often converge even at an illegal candidate. Here, we demonstrate an analogue electronic computing system for solving the travelling salesman problem, which mimics efficient foraging behaviour of an amoeboid organism by the spontaneous dynamics of an electric current in its core and enables a high problem-mapping flexibility and resilience using a resistance crossbar circuit. The system has high application potential, as it can determine a high-quality legal solution in a time that grows proportionally to the problem size without suffering from the weaknesses of Ising machines.

## Introduction

Combinatorial optimization problems are computationally demanding tasks appearing in various practical applications, such as optimization of traffic flow, path planning, nurse scheduling and advertisement allocation^1–4^. Often these problems become intractable for conventional von Neumann-type computers, such as general-purpose CPUs; they need to evaluate an enormous number of candidate solutions in a serial manner, where the number of the candidates grows exponentially with the problem size, leading to “combinatorial explosion”.

The travelling salesman problem (TSP) is one of the most widely investigated combinatorial optimization problems; given a map of $N$ cities, the TSP is stated as a problem of finding the shortest route for visiting each city exactly once and returning to the starting city^5,6^, where the number of all legal solutions (possible routes) grows factorially as $\left(N-1\right)!/2$. The TSP is a nondeterministic polynomial time (NP)-hard problem, that is, any exact algorithm to find the optimal solution (exactly the shortest route) for a general instance in polynomial time is not known so far. On the other hand, various nature-inspired approximation algorithms have been proposed to promptly derive a high-quality legal solution (a satisfactory short route), such as the k-opt algorithm with simulated annealing, genetic algorithm, particle swarm optimization algorithm and ant colony optimization algorithm^7–11^.

Many of the nature-inspired algorithms are formulated to update multiple variables in parallel to achieve rapid search, whereas the serial process of the CPU that manipulates a single bit at a time can only simulate the parallelism in a limited way. Therefore, it is required to develop a novel domain-specific computing architecture to implement these algorithms to maximize their parallel search capabilities by exploiting physical processes of specific hardware, expecting to cultivate new potentials and markets of combinatorial optimization. The first physical computing system for solving the TSP comprises the Hopfield’s recurrent neural network implemented by an electronic circuit^12,13^. This system, however, was not so useful because it frequently converges at a local minimum state (a low-quality solution) and sometimes cannot reach even a candidate solution for some problem instances^14–16^. In fact, for some instances of 10-city TSP, it was reported that the rate of finding a legal solution was at most 20%^15^.

**Figure 1:**
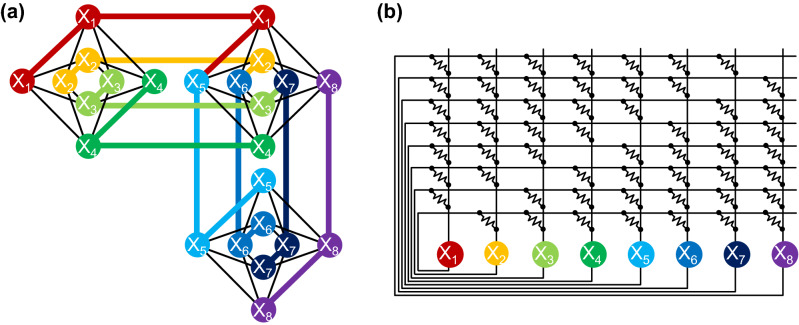
Physical mapping schematics of the TSP to (**a**) the Ising machine and (**b**) electronic amoeba. Each state variable ${X}_{i}$, taking a value of 1, determines where and when should a salesman visit. (**a**) Chimera graph for fully connecting an arbitrary pair of spin variables ${X}_{i}$ and ${X}_{j}$, requiring redundant variables for each ${X}_{i}$. (**b**) Resistance crossbar of the instance-mapping circuit (IMC), achieving full connectivity without introducing any redundant variables.

In recent years, many proposals on rapid combinatorial optimization by physically implementing the “Ising machine” or “annealing machine” with several exploration mechanisms, such as quantum annealing (QA) and related methods, have attracted a lot of public attention^17–21^. Each of these machines explores an optimal solution by mapping the problem to a process of finding a minimum-energy spin assignment in the Ising model that abstracts a ferromagnetic material^17,22,23^. However, the problem mapping and parameter tuning of the Ising model are complex and costly. For the $N$-city TSP, the regular layout of spin variables with sparse connectivity requires redundant variables to be introduced in the order of ${N}^{4}$ to handle irregularly distributed cities, leading to a rapid increase in the circuit area^24–28^. Figure 1a shows a graph structure of the Ising model, which is referred to as a chimera graph^17^. In such a structure, the consistency between the redundant variables can be broken potentially. When the parameter tuning cannot be made appropriately, the Ising model sometimes converges at an illegal state in which constraints of the TSP to exclude the revisiting of a once-visited city and to exclude simultaneous visits to multiple cities are violated (see Supplementary Information [SI]). Actually, in the physical Ising machines solving the Max-cut problem, the probability of reaching a legal state varies depending on the connectivity among graph nodes, and the sparsely connected graph often results in even worse performance owing to the variable overhead^29^.

**Figure 2:**
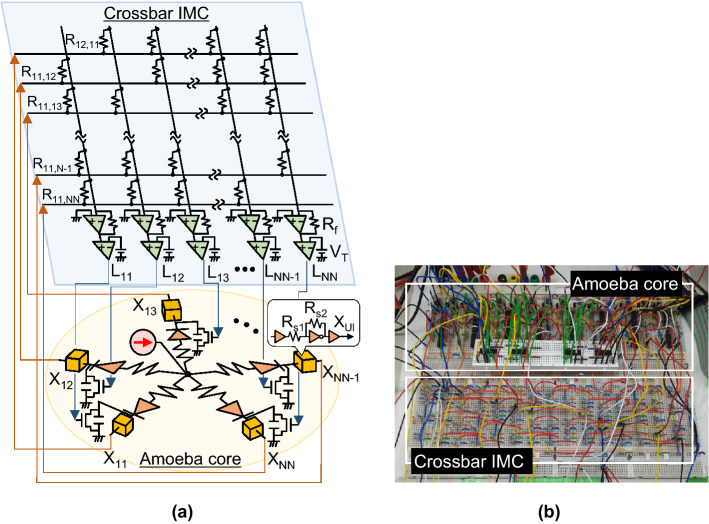
Electronic amoeba for solving the TSP. (**a**) Schematic of the system consisting of the amoeba core and IMC. For solving the $N$-city TSP, the amoeba core uses ${N}^{2}$ units to represent state variables, each of which performs the charging and discharging dynamics of the capacitor to express expanding and shrinking behaviour of a pseudopod-like branch of the amoeba. The IMC executes product-sum operations of recurrent neural network dynamics for the bounceback control to govern the interactions among the variables (see “Methods”). (**b**) Photo of fabricated electronic amoeba consisting of commercially available CMOS devices.

Our approach is not based on the Ising model. We focus on a living amoeboid organism that performs trial-and-error behaviour to survive efficiently and resiliently in a harsh environment, deforming its gel-like body^30,31^. Here, we demonstrate, as a proof of concept, an analogue electronic computing system called an “electronic amoeba”^32,33^, inspired by the food search and risk avoidance behaviour of a single-celled amoeboid organism, *Physarum polycephalum*^30,31,34–39^. In the electronic amoeba, an arbitrary TSP instance can be mapped on the resistor network of a crossbar structure shown in Fig. 1b, which we call the “instance-mapping circuit (IMC)”. The architecture of the IMC is similar to that of the Hopfield’s recurrent neural network^12,13^. However, as shown in Fig. 2a, it is connected with the “amoeba core”, which contributes to avoiding the convergence at an illegal state. In the author’s previous works^35,38,39^, we formulated an algorithm representing a primitive idea of the electronic amoeba and predicted its potential performance without any physical implementation. In this paper, with results obtained from numerical simulations using a conventional computer and laboratory experiments using a physically fabricated circuit (Fig. 2b), for the first time, we show that the electronic amoeba finds a high-quality TSP solution in a time that is proportional to $N$. The electronic amoeba is highly scalable and energy-efficient as it comprises existing complementary metal-oxide semiconductor (CMOS) devices and is expected to be useful for wide range of applications.

## Results

Figure 2a shows a schematic of the electronic amoeba composed of the amoeba core and IMC, which electronically mimics the solution-searching dynamics of a so-called “amoeba-based computer” that employs a living amoeba to search for a solution to the TSP^38,39^ (see Supplementary Information). The state of each unit in the amoeba core represents the decision on where and when to visit. The IMC implements a type of feedback control, called the “bounceback control”, which refers to the TSP constraints and intercity distances from the given map and sends a “bounceback signal” to each unit in the amoeba core^36–39^; the signal is determined in accordance with the recurrent neural network dynamics defined by Eq. (1) in “Methods”.

**Figure 3:**
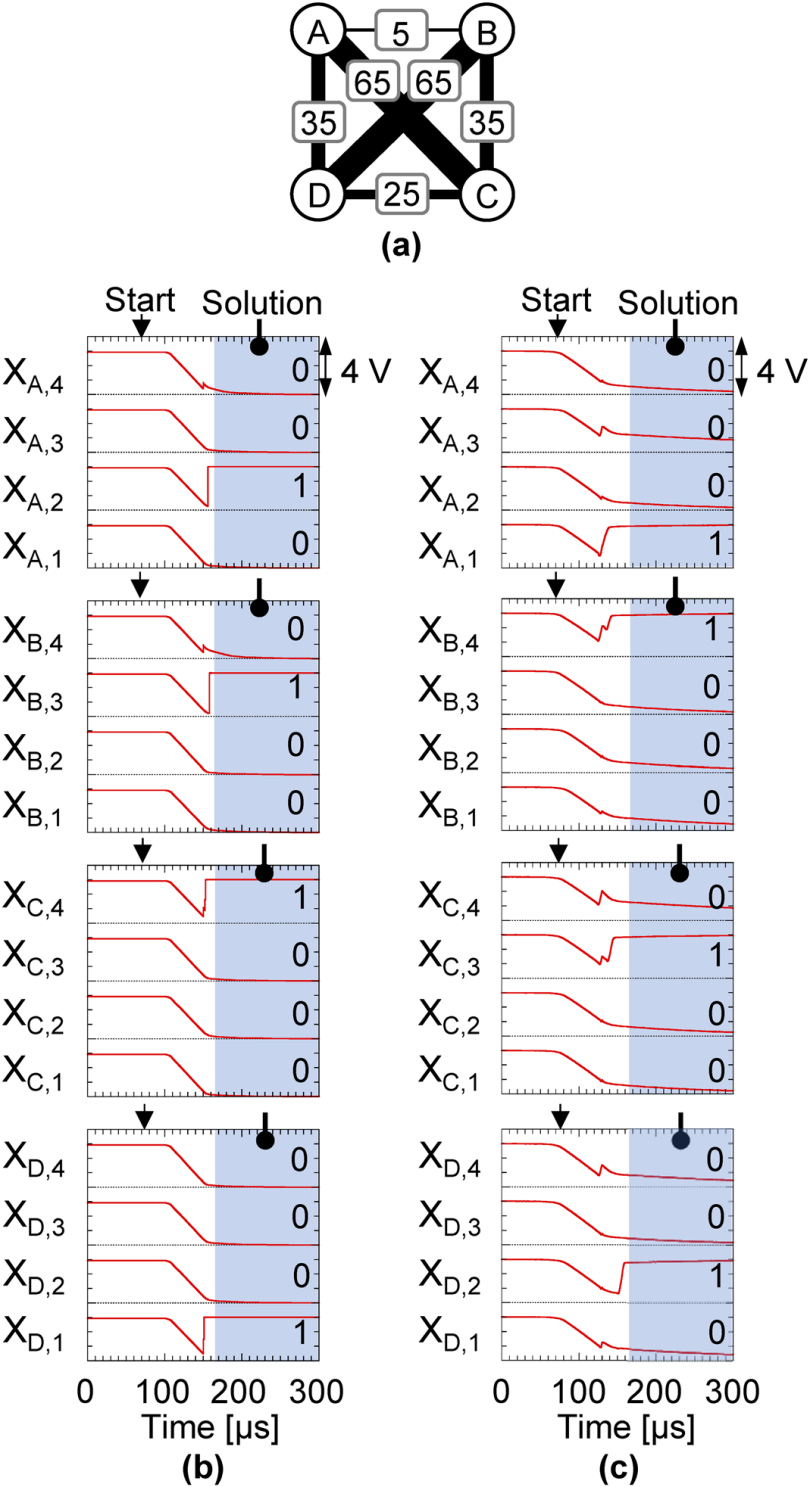
Solution-searching dynamics of electronic amoeba. (**a**) 4-city TSP instance. Each edge weight indicates the distance between corresponding cities. (**b**,**c**) The dynamics of state variables in the circuit simulation and fabricated system, respectively. Variable ${X}_{V,k}$ represents that city $V$ is visited at the $k$th order. The shortest routes, *D → A → B → C → D* and *A → D → C → B → A*, were found by the simulation and fabricated system, respectively.

We first conduct numerical simulations using a circuit simulator run on a conventional computer (see “Methods”) to determine if the electronic amoeba can solve the 4-city TSP instance shown in Fig. 3a. Figure 3b demonstrates an example of output waveforms obtained from the circuit simulation. The subscripts of the state of each unit ${X}_{V,k}$, $V$ and $k$, mean that city $V$ is visited at the $k$th order. Initially, every unit takes a state of 1 because the capacitor charge is set to zero. The IMC then sends the bounceback signals to all units to flip their states from 1 to 0, as the all-one states are violating constraints of the TSP. The state of each unit gradually approaches to 0 when charging the capacitor by injecting a current from the current source. After several flips have been induced by the bounceback control, the dynamics of all units became stable as shown in the hatching area in Fig. 3b. At this moment, the electronic amoeba finds an optimal solution, *D → A → B → C → D*, which corresponds to the shortest route.

**Figure 4:**
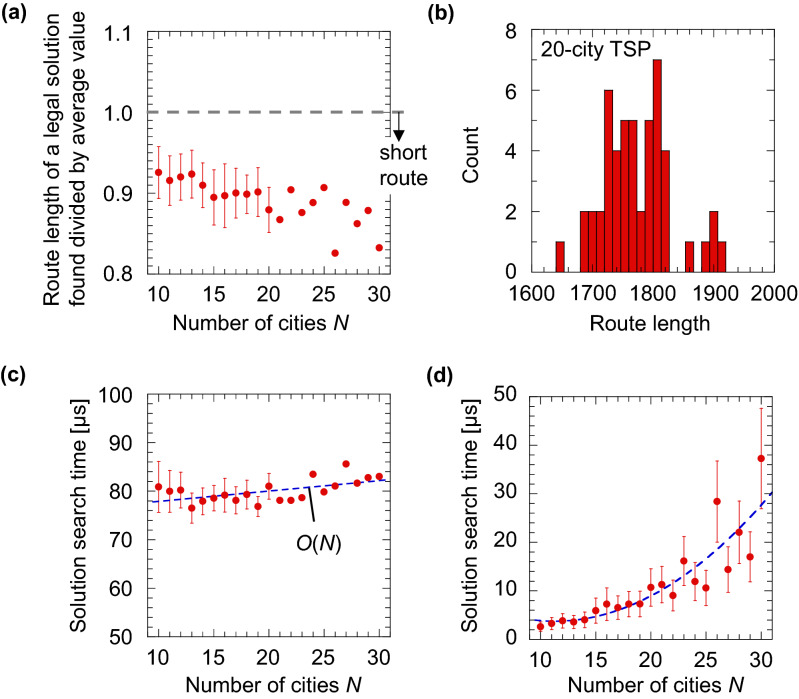
Solution-searching performance of circuit-simulator-based electronic amoeba and 2-opt evaluated as a function of the problem size. (**a**–**c**) The results obtained from the electronic amoeba and (**d**) from 2-opt. (**a**) Route length normalized by the average length calculated by random sampling. Error bars for the 10–20-city instances denote standard deviations obtained from 50 trials, whereas those for 21–30-city cases are not shown as only a trial was performed for each case. (**b**) Histogram of route lengths of the solutions found for a 20-city instance after 50 trials. The average route length calculated by random sampling was 2016, whereas that obtained by the electronic amoeba was 1780. (**c**) Solution search time estimated from the dynamics generated by the circuit simulator (see “Methods”). (**d**) Solution search time required for the calculation using a conventional computer (see “Methods”).

Using a circuit simulator, we have investigated the solution-searching performance of the electronic amoeba for $N$ ranging from 10 to 30. We performed 50 trials for each instance for $N$ not greater than 20 but only once for each $N$ more than 20 because the simulation time increased rapidly; it took 5 h for the 20-city instances but took 6 days for the 30-city cases. In each trial, resistances in the units were randomly assigned from 1 Ω to 10 kΩ to lead the electronic amoeba to explore a wider state space. To evaluate the rate of finding a legal solution in the electronic amoeba, we performed 560 trials for solving the TSP of 10–30 cities. The rate was found to be 100%; the system certainly converged to one of the legal solutions for every try. This is because the amoeba core always stabilizes at a steady state in which no variable violates the TSP constraints^39^; in such a state, no further change in all units in the amoeba core is induced by the bounceback signals. Figure 4a shows the length of the route obtained by the circuit simulator. In Fig. 4a, the vertical axis is normalized by the average route length obtained from random sampling of 10,000 trials; if a value on the vertical axis is less than 1.0, it implies that the quality of the legal solution found is higher than that found by random sampling. The results indicate that the electronic amoeba finds higher-quality solutions than random sampling. Moreover, the average of the route length was on a declining trend; the quality did not degrade even when the problem size $N$ became larger. By introducing random variations in the resistances of the units, each unit varies the velocity of the transition from state 1 to 0, and a wide variety of legal solutions were found as shown in Fig. 4b; it reached different solutions even for an identical instance, although it did not guarantee to reach the optimal one.

Figure 4c shows that the average time required for the electronic amoeba to find a legal solution increases almost only linearly as a function of $N$. This result reproduces the observation confirmed in the amoeba-based computer well; the living amoeba reached an approximate solution of the TSP in linear time^38,39^. A hypothetical mechanism of the linear-time solution has been proposed with an abstract mathematical model of the solution-searching dynamics of the amoeba-based computer; the numerical simulation of the model, named “AmoebaTSP”, suggested that the linear-time solution can be achieved if the hub of the single-celled organism could supply the intracellular resources to grow its branches with a constant rate, even while responding to the bounceback signals^38,39^. The linear-time operation is attributed to the design of the bounceback control together with parallel operations of all units in the amoeba core. The units try to choose a path between two cities having a shorter distance, according to information on shortness of the distances of possible paths through accumulating and comparing experiences of being inhibited by the bounceback signals; the two units representing a shorter-distance path are less frequently inhibited compared to the others and are allowed to take relatively larger state values. The bounceback rule is designed so that once a path and their visiting orders are decided, the rule restricts the amoeba core from changing the decision after that. Thereby the system decides each path one by one, avoiding illegal paths.

The electronic amoeba will reach a high-quality solution in linear time even for larger-size instances if it follows a similar mechanism as AmoebaTSP. Moreover, the solution search time of the electronic amoeba can be reduced further as the flipping of the state of each unit can be accelerated by increasing the current and/or decreasing the capacitance (see Supplementary Information).

We compared the solution-searching performance of the circuit simulator-based electronic amoeba with that of a representative stochastic local search algorithm, the 2-opt (see “Methods”), which is a simple and fast method requiring no parameter optimization^7^. The quality of the solution obtained by the 2-opt gets higher (and saturates eventually) as its main operation iterates for a larger number of times, but we terminated the iteration when the quality become equal to that obtained by the electronic amoeba and measured the time required by the termination. As shown in Fig. 4d, the 2-opt required an amount of time that grows as a quadratic function of the problem size, whereas the electronic amoeba needed only linear time to present the solution with the same level of quality (Fig. 4c). Accordingly, the electronic amoeba, once physically implemented, will be more useful for finding a high-quality solution in a shorter search time than the 2-opt when run on a conventional computer and when the number of cities exceeds 50.

**Figure 5:**
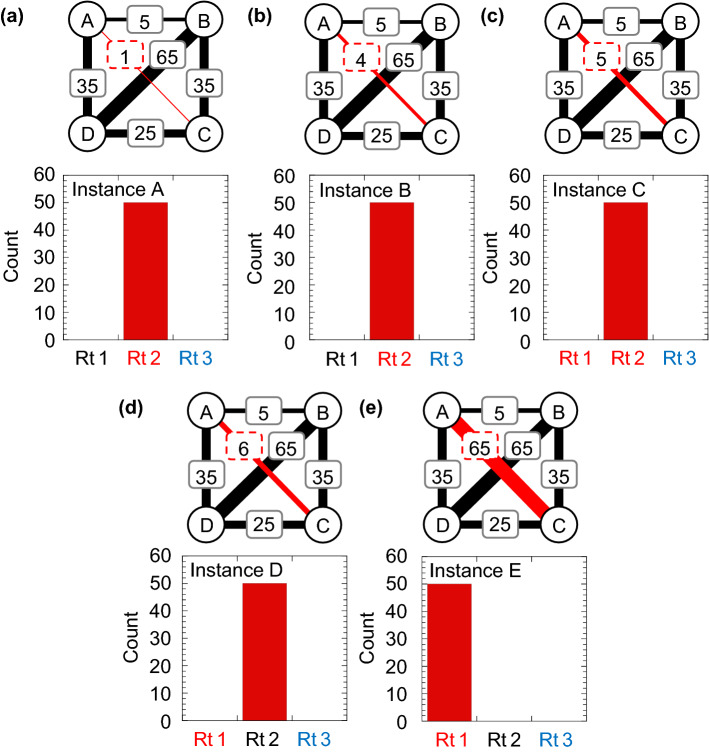
Results obtained from the fabricated electronic amoeba for 4-city TSP instances that gave three possible routes (legal solutions). (**a**–**e**) Histograms of the routes obtained from 50 trials. Abbreviations in horizontal axes are Rt 1: *A → B → C → D → A*, Rt 2: *A → C → D → B → A* and Rt 3: *A → D → B → C → A*.

By fabricating the electronic amoeba physically (Fig. 2b) using CMOS devices, we have verified that the fabricated system can solve various 4-city TSP instances as shown in Fig. 5a–e, where the optimal and worst solutions are summarized in Table 1. The amoeba core comprises 16 branches, and we can map an arbitrary 4-city instance by changing the resistance values in the IMC. The time evolution of the state variables in the fabricated system is shown in Fig. 3c. At an initial stage, the variables behaved similarly to those in the circuit simulation (Fig. 3b), and they became stable after reaching a solution. The bottom of Fig. 5a–e shows that the system found the shortest route for instances A–C and E where we performed 50 trials for each instance without changing the resistance values.

**Table 1:** Summary of TSP instances and their route lengths.

	Instance A	Instance B	Instance C	Instance D	Instance E
Rt 1: A → B → C → D → A	100	100	100	100	100
Rt 2: A → C → D → B → A	96	99	100	101	160
Rt 3: A → D → B → C → A	136	139	140	141	200

The fabricated system could not find the shortest route for instance D as shown in Fig. 5d, although the shortest route length of D equals that of C. This would be attributed to a number of device variations in the fabricated circuit, such as the threshold voltage variation in the CMOS inverter, offset voltage variation in the operational amplifier and difference in the wiring length in the IMC, which might create a preference when making a decision. However, for the cases where the route lengths are widely distributed such as instance E, the system reached the optimal solution, overcoming the preference. When solving larger-sized instances and introducing more noise, the system performance is expected to become reliable and robust (Supplementary Fig. S6), because the differences in qualities (route lengths) of legal solutions of those instances become relatively smaller than that of smaller-sized instances, depending on noise amplitude.

Note that it is necessary for the Ising machines to expend considerable efforts on problem-mapping and parameter-tuning processes prior to solving the problem. If this pre-processing could be made properly, the Ising model combined with simulated annealing exhibits a better performance in finding a higher-quality TSP solution than the electronic amoeba, otherwise it fails to reach even a legal solution (see Supplementary Information). In contrast, there is no need for the electronic amoeba to execute complex and costly pre-processing because the IMC offers a high problem-mapping flexibility with its unrestricted connectivity between an arbitrary pair of variables and requires only simple numerical operations to determine the parameters (see “Methods”). Moreover, when the resistors in the IMC are replaced with memristors or atomic switches, enabling dynamic resistance rewriting^40–44^, the TSP instance to be solved can be updated promptly even in the middle of the solution-searching process, and the bounceback control will enable the amoeba core to find a new solution of the updated instance effortlessly by slightly revising the previous one. Such a dynamically rewritable IMC will enable the electronic amoeba to respond resiliently to sudden changes in the problem constraints caused by unexpected failures occurring in ever-changing practical situations.

The scalability is an important issue for the implementation of the electronic amoeba. For solving $N$-city TSP, the amoeba core needs ${N}^{2}$ units, and the crossbar IMC requires $2{N}^{2}$ wires and ${N}^{4}$ resistors at cross points of the wires. The circuit area of the electronic amoeba, therefore, grows in the order of ${N}^{2}$, which is less costly than the Ising machines that require the area in the order of ${N}^{4}$. This order is apparently comparable to that of the Ising model-based digital machines with full connectivity in logical level. However, the physical crossbar in the electronic amoeba produces advantages in terms of execution time and energy consumption; the crossbar allows to execute the bounceback control for all variables in a fully parallel manner, whereas the FPGA-based digital Ising machine requires a lot of memory accesses to achieve the logical full-connectivity, consuming higher time and energy costs. Owing to the modern digital LSI technology, each capacitance in the amoeba core will be downsized to the minimum level where its charging time is distinguishable from that of the parasitic elements. It is expected that state-of-the-art nanotechnology to fabricate a nanoscale crossbar structure equipped with memristors to represent analogue resistance values^42,43^ will suppress the increase in the physical size of the IMC. The electronic amoeba can be built and maintained with a significantly cheaper cost than the Quantum annealing machine that requires a lot of elaborate equipment for refrigeration and maintenance of quantum coherence. Furthermore, while the computing speed of other Ising machines are unscalable as they suffer from the "Neumann bottleneck" that limits the data transfer rate in a data bus between memory and operation sections, the electronic amoeba does not encounter such a limitation.

In this paper, we demonstrated that, given an arbitrary TSP instance, the electronic amoeba enables to start the computing readily after simple resistance determination operations in the IMC and to find a high-quality solution certainly in only linear time, exploiting the spontaneous dynamics of the electric current in the amoeba core. This reliable and swift solution-searching capability could be beneficial for particular applications that prioritize the search time over the quality of a solution found. For example, in a situation at a disaster site where presenting reliable evacuation routes for residents is necessary, making a swift announcement should be prioritized than deriving the exactly optimal routes. The electronic amoeba would be more useful than using conventional computers to run the stochastic local search algorithm when the number of cities exceeds a hundred or more. Moreover, the compactness of the IMC suggests that the system-on-chip approach supported by semiconductor LSI technologies will further enhance the scalability and energy-efficiency, making it useful for wider cloud- and edge-computing applications. One of our future subjects is to improve the quality of the solution found by the electronic amoeba. Possible approaches are to assign appropriate initial states to the amoeba core units using the genetic algorithm, to impose stochastic fluctuations using a hardware random number generator to forcibly escape local minimum solutions (see SI), and to introduce delays in the bounceback control to induce the oscillation of state variables^45^.

## Methods

### Bounceback control of the electronic amoeba

To implement the bounceback control for solving the TSP on the crossbar IMC, we followed the basic scheme of the amoeba-based computing system (see SI)^36–38^. For the $N$-city TSP, $N\times N$ state variables are needed where each variable ${X}_{Vk}$ with subscript $Vk$ indicates that city $V$ is visited at the $k$th order: when ${X}_{Vk}=1$, the city $V$ is visited at the $k$th order. The bounceback signal in the electronic amoeba is generated by the crossbar IMC shown in Fig. 2a. The IMC evaluates all bounceback signals in a parallel and continuous fashion. The IMC circuit computes the bounceback signal is as follows,

1 $$\begin{array}{c}{L}_{Vk}=F\left(\sum_{Ul}\frac{{R}_{f}}{{R}_{Vk,Ul}}\cdot {X}_{Ul}-{V}_{T}\right),\end{array}$$

where ${R}_{f}$ is a feedback resistor for the op-amp and ${R}_{Vk,Ul}$ is a resistor at cross point of the output line $Vk$ and the input line $Ul$ in the crossbar corresponding to the intercity distance. ${V}_{T}$ is a threshold value of the threshold function $F$ in the output portion of the crossbar IMC, which is implemented by a comparator. In this study we used ${V}_{T}=1.5$ V and ${R}_{f}=10$ kΩ. ${R}_{f}/{R}_{Vk,Ul}$ is given by

2 $$\begin{array}{*{20}c} {\frac{{R_{f} }}{{R_{{Vk,Ul}} }} = \left\{ {\begin{array}{ll} {0.5} \\ {{\text{dist}}\left( {V,U} \right)/\lambda } \\ 0 \\ \end{array} \begin{array}{ll} \quad {({\text{if }}V = U{\text{at }}k \ne l{\text{or }}V \ne U{\text{at }}k = l)} \\ \quad {({\text{if }}V \ne U{\text{and }}\left| {k - l} \right| = 1)} \\ \quad {({\text{otherwise}})} \\ \end{array} } \right.} \\ \end{array}$$

where $\text{dist}(V,U)$ is an intercity distance between cities $V$ and $U$ and $\lambda$ is a normalization factor. ${R}_{f}/{R}_{Vk,Ul}$ defines the interaction between the state variables so as to (1) prohibit the system from visiting the city once visited, (2) prohibit the system from visiting different cities at once, and (3) minimize the total length of a travel route. $\lambda$ is a normalized coefficient and we use $\lambda =\text{max}(\text{dist}({V}^{^{\prime}},{V}^{{^{\prime}}{^{\prime}}})+\text{dist}({V}^{{^{\prime}}{^{\prime}}},{V}^{{^{\prime}}{^{\prime}}{^{\prime}}}))/\theta$, where $\theta$ is a threshold in the sigmoid function and $\text{dist}({V}{^{\prime}},{V}{{^{\prime}}{^{\prime}}})+\text{dist}({V}{{^{\prime}}{^{\prime}}},{V}{{^{\prime}}{^{\prime}}{^{\prime}}})$ is the maximum value of the total distance between the cities ${V}^{^{\prime}}$ and ${V}^{{^{\prime}}{^{\prime}}{^{\prime}}}$ via ${V}^{{^{\prime}}{^{\prime}}}$.

A CMOS inverter with resistors shown as square boxes in Fig. 2a represents a sigmoid function. Here, the slope of the sigmoid function is defined by the resistance values ${R}_{s1}=390$ kΩ and ${R}_{s2}=2.2$ MΩ. The measured behaviour of a pseudopod-like branch in the fabricated amoeba core is shown in Supplementary Information.

The bounceback control ensures the system does not stabilize whenever there are constraints that remain unsatisfied as the bounceback signals flip the constraint-violating variables from 1 to 0. The IMC does not have any free parameters, although the degree of resistance variation in the amoeba core is adjustable. The electronic amoeba, therefore, does not require any complex and costly pre-processing for problem mapping and parameter tuning.

### Circuit simulator

We used the electronic circuit simulator, LTspice XVII (Simulation Program for Integrated Circuit Emphasis simulator, SPICE, https://www.analog.com). The current source is $5{N}^{2}$ µA for the number of cities $N$, capacitance $C$ is 500 pF and initial voltage of capacitors is set to 1.5 V in the circuit simulation. In the fabricated electronic amoeba integrating the commercial discrete devices, $C$ is the sum of discrete components near 470 pF and the current source is set to 80 µA.

### 2-opt

The stochastic local search algorithm, 2-opt, updates the route by iterating the following main operation starting from a randomly chosen route (a legal solution); it chooses two cities randomly and inverts a path between the two locally in the current route if the inversion resulted in a reduction in route length. We performed the 2-opt calculation using a conventional computer (Intel Xeon processor E5-1650 v2 @ 3.50 GHz).

## Supplementary information

Supplementary Information.
