# Supplementary material for: Amoeba-inspired analog electronic computing system integrating resistance crossbar for solving the travelling salesman problem
Source: Sci Rep. 2020 Nov 27;10:20772. doi: 10.1038/s41598-020-77617-7 (PMC7695837; doi:10.1038/s41598-020-77617-7)
Supplement: Supplementary file 1 — Supplementary Information. [file 41598_2020_77617_MOESM1_ESM.pdf]

Supplementary Information for

Amoeba-inspired Analog Electronic Computing System Integrating Resistance Crossbar for Solving the Travelling Salesman Problem

Kenta Saito, Masashi Aono, and Seiya Kasai

Contents

1. Overview of the amoeba-inspired computing paradigm

2. Dependence of solution-searching performance of the electronic amoeba on circuit parameters

3. Approaches to improve the solution quality of the electronic amoeba

4. Solution-searching performance of the Ising models

1. Overview of the amoeba-inspired computing paradigm

1.1 Amoeba-based computing system

Figure S1(a) depicts the basic scheme of an amoeba-based computing system, called the "bounceback control"[38]. The amoeba-based computing system is constructed with two components. The first is a single-celled amoeboid organism, a plasmodium of the true slime mould *Physarum polycephalum* (hereafter "amoeba" for simplicity), placed inside a stellate container that has several lanes (grooves), each of which enables the amoeba to expand and shrink its pseudopod-like branch to indicate a value of each corresponding state variable. The second is a controller that monitors the shape of the amoeba, governs the interactions between the variables in accordance with certain rules and irradiates visible light on a number of branches of the amoeba to induce their shrinking behaviour.

The amoeba on an agar plate optimizes its body shape to maximize its body area for maximal nutrient absorption while minimizing the risk of being illuminated by light, where the volume of the amoeba in the container is almost conserved. When the amoeba expands its branch in lane i so that the proportion of the area covered by the branch exceeds a threshold, state variable x_i takes a value of 1, otherwise 0. The controller always monitors the amoeba branches and determines which branches are to be flipped from 1 to 0 in accordance with certain rules. Once the controller illuminates a branch to be flipped, the branch gradually shrinks due to its photoavoidance response. This system reaches a legal solution when the light illumination pattern and the shape of the amoeba become stable. Note that the expanding and shrinking behaviour of each amoeba branch to the light fluctuates stochastically; with a small probability, the branch expands even under illumination and hesitates to expand even under the non-illuminated condition. The fluctuation is found to play an important role in exploring a wider state space[33,36,37].

In the amoeba-based computing system for solving N-city TSP, the variable $X_{Vk}(t) \in$

[0.0,1.0] is represented by the ratio of the area where a pseudopod of the amoeba fills the lane Vk at time t. The pseudopod shrinks when it is illuminated by visible light due to the photoavoidance. $L_{Vk}(t) \in [0.0,1.0]$ is the bounceback signal involving the illumination condition of lane Vk at t; $L_{Vk} = 1.0$ suggests the illumination with the maximum intensity, whereas $L_{Vk} = 0.0$ suggests no illumination. The bounceback rule for the TSP is derived from the modified formulation developed for mapping on the Hopfield's recurrent neural network[12,13]. The bounceback signal is defined as follows:

$$L_{Vk}(t + \Delta t) = 1 - \sigma_{\gamma_1,\eta_1}\left(\sum_{Ul} W_{Vk,Ul} \cdot \sigma_{\gamma_2,\eta_2}\big(X_{Ul}(t)\big)\right), \quad \text{(S1)}$$

$$\sigma_{\gamma,\eta}(x) = \frac{1}{1 + \exp\big(-\gamma \cdot (x - \eta)\big)}, \quad \text{(S2)}$$

where $W_{Vk,Ul} = -R_f/R_{Vk,Ul}$. Equations (S1) and (S2) determine whether the lane Vk is illuminated or not. For example, when $L_{Vk} \geq 0.5$, the branch Vk is illuminated and the pseudopod shrinks, otherwise the branch is not illuminated, and the pseudopod expands. Parameters γ and η in equation (S2) correspond to the inverse temperature in statistical mechanics and threshold value, respectively. In the amoeba-based computing system[38], the parameters were set as follows: $\gamma_1 = 1000$, $\eta_1 = -0.5$, $\gamma_2 = 35$, and $\eta_2 = 0.6$. It is noted that any other parameter optimization is not necessary[38]. In the case of the electronic amoeba, we use a step function instead of the sigmoid function $\sigma_{\gamma_1,\eta_1}(x)$ in the crossbar IMC, since the sigmoid function is the almost same as a step function when γ is large enough.

The single-celled organism in the amoeba-based computing system attempts to find a shape where the pseudopods in N branches fully elongate along the non-illuminated lanes by accumulating the history of the illumination. In accordance with the policy of the bounceback control, the amoeba shape representing the shortest route, that is the optimal solution, corresponds to the most stable state. At this state the amoeba body area is maximized, where nutrient absorption is maximized and the risk of illumination is minimized[35].

1.2 Amoeba-inspired electronic computing system

Figures 2(a) and S1(b) shows a schematic of the electronic computing system for solving the travelling salesman problem (TSP), called an electronic amoeba[32,33], which we designed on the basis of a mathematical model of the solution-searching dynamics of the amoeba-based computing system, AmoebaTSP, formulated by Aono *et al*[35,38]. In the amoeba core, the current flowing into unit i represents the expanding behaviour of the branch, and state variable x_i take a value of 1, otherwise 0. Key attributes of the solution-searching dynamics of the system are the conservation of the volume of the amoeba, photoavoidance response and stochastic fluctuation. The volume conservation is represented by the Kirchhoff's current law in the network hub. The flip of the variable is carried out by the control of a current flow of the field-effect transistor (FET) in accordance with the bounceback signal sent from the crossbar instance-mapping circuit (IMC). The stochastic fluctuation is mimicked by imposing noise to the FET in the branch[33]. In this study, although we did not implement any external fluctuation source on the system intentionally, we found that the system intrinsically generates a fluctuated behaviour in its dynamic operation.

Figures S2(a) and S2(b) show the circuit of the pseudopod-like branch and the experimentally measured response, respectively. When the MOSFET was turned off, corresponding to $L_{Vk} = 0$, the capacitor charged the current from the current source and the output of the sigmoid-like function circuit gradually approached low level. On the other hand, when it was turned on, corresponding to $L_{Vk} = 1$, the capacitor discharged and the output immediately became high level.

The configuration in the IMC for solving the TSP appears to be similar to that of the Hopfield's recurrent neural network[12,13]. However, the interactions between the variables in the electronic amoeba are derived from a different formulation; in the bounceback control, the interaction is defined to prohibit state variables from violating the constraints of the TSP to

exclude revisiting a once-visited city and to exclude simultaneous visits to multiple cities[38].

Aono *et al*. formulated another algorithm for solving the Boolean satisfiability problem (SAT), named AmoebaeSAT[36,37], and implemented it on analogue and digital electronic circuits to maximize their rapid search performance[38,39]. These electronic amoebae for solving the TSP and SAT can easily handle arbitrary problem instances without any costly pre-processing, and their dynamics stabilize only at legal solutions that satisfy all given constraints. As electronic amoebae can be composed of conventional complementary metal-oxide semiconductor (CMOS) devices, they are highly scalable, energy-efficient, and suitable for both cloud- and edge-computing applications.

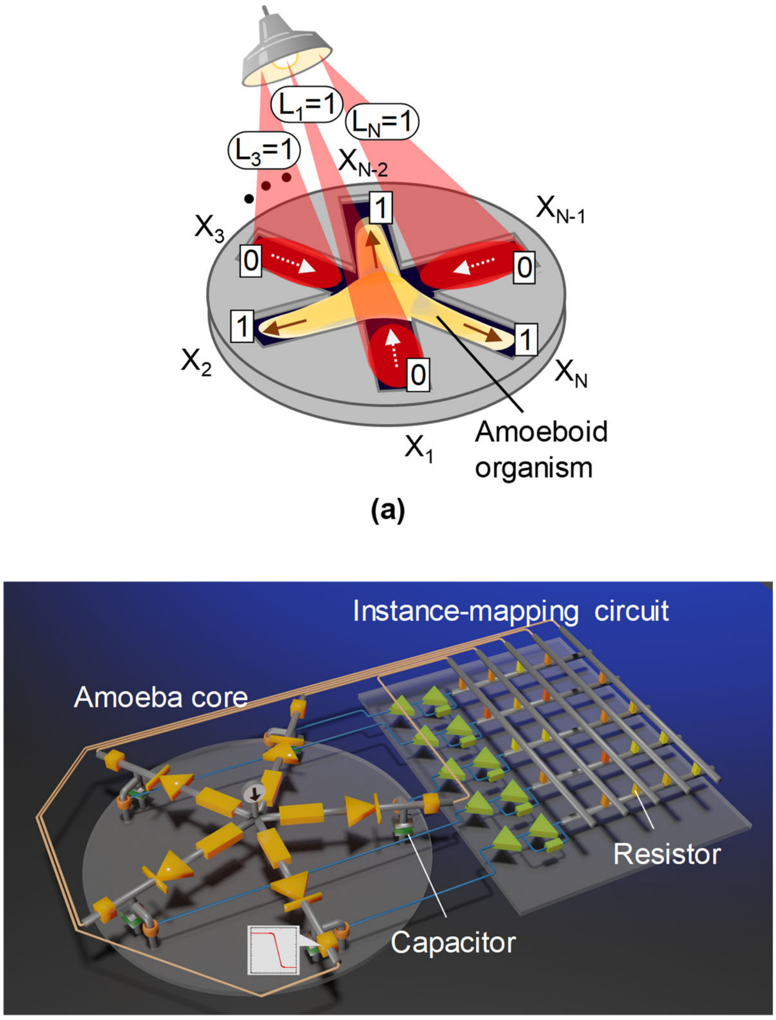

(a)

(b)

Figure S1 (a) Basic scheme of the amoeba-based computing system. The amoeboid

organism expands its pseudopod-like branches. However, the branch shrinks when illuminated by light. The solution-searching of the amoeba-based computing system progresses with the changes in the amoeba shape and illumination pattern. (b) Circuit diagram of the electronic amoeba for solving the TSP.

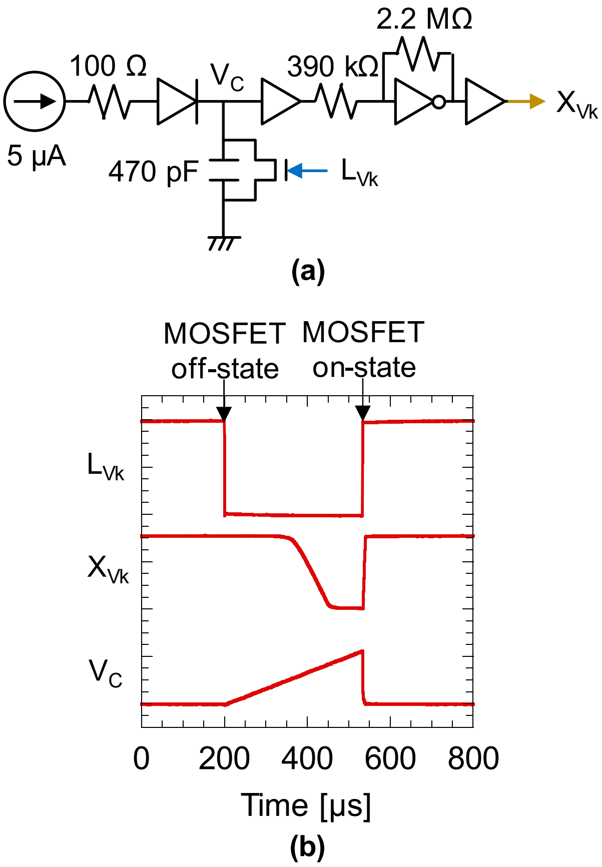

Figure S2 A pseudopod-like branching unit in the electronic amoeba. (a) Circuit diagram and (b) measured response and voltage of the capacitance in a fabricated circuit.

2. Dependence of solution-searching performance of the electronic amoeba on circuit parameters

In the electronic amoeba, the transition time of the state variable between two states, 1 and 0, depends on the current injected from the current source and the capacitance value in each unit. The solution search time depends on these parameters. We confirmed this point by conducting 50 trials of the circuit simulation for a 10-city TSP instance, where the resistance values in the units were randomly assigned. Figures S3(a) and S3(b) are the obtained solution search times and route lengths as a function of the hub current, respectively. Those of the capacitance dependencies are shown in Figs. S3(c) and S2(d), respectively. The solution search time was decreased either by increasing the current or by decreasing the capacitance. The results suggest that the solution search time is scaled inversely to the hub current and linearly to the capacitance. On the other hand, the solution quality was slightly degraded when the solution search time was reduced as shown in Figs. S3(b) and S3(d). This is because the variation effect of the resistance increases relatively when the current is increased and/or the capacitance is decreased. Therefore, we can avoid the degradation of the solution quality by reducing the resistance variation.

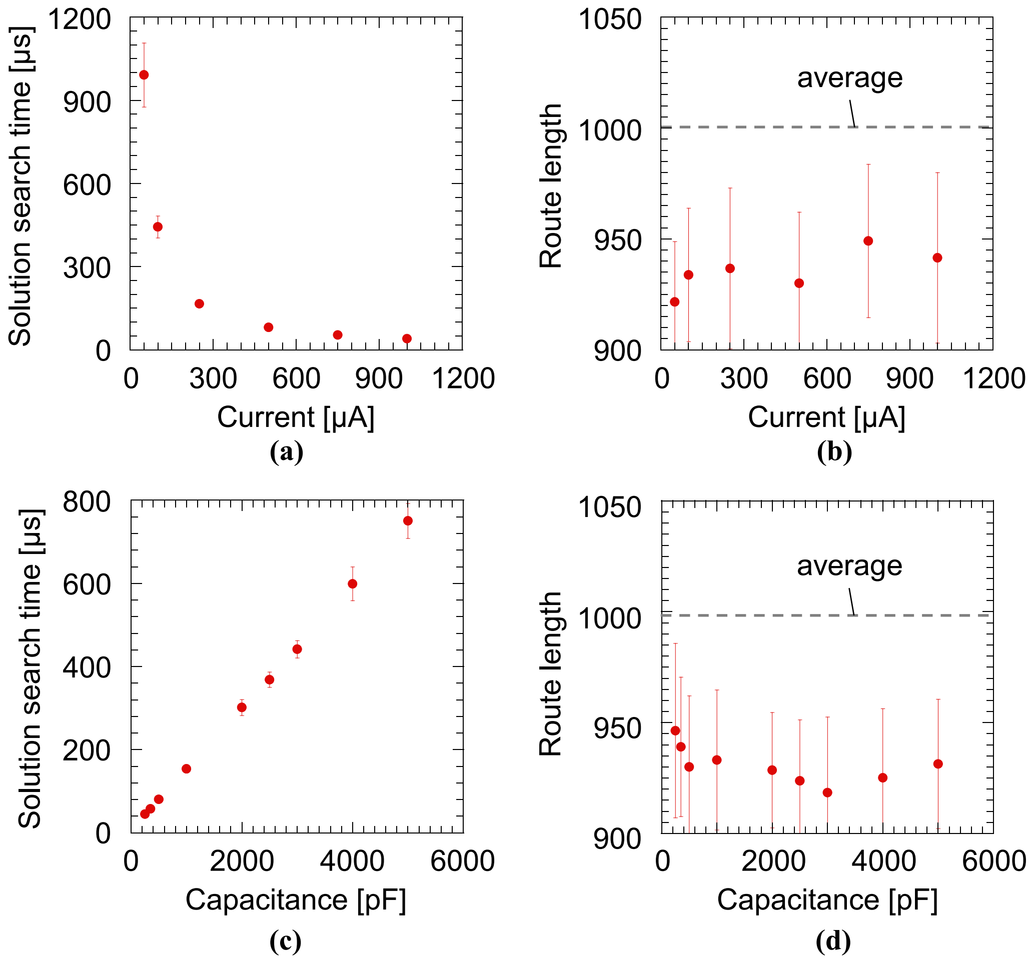

Figure S3 Dependence of solution search time and quality on current and capacitance evaluated using a circuit simulator. (a) and (b) show the results of the solution search time and quality as a function of the current in the hub of the amoeba core, respectively. (c) and (d) show the results of the time and quality as a function of the capacitance in the unit, respectively. The solution search time was reduced by increasing the injected current or decreasing the capacitance.

3. Approaches to improve the solution quality of the electronic amoeba

3.1 Amoeba-GA hybrid approach

As a possible approach to improve the quality of the solution found by the electronic amoeba, we have investigated the dynamic adjustment of the resistance variation in the amoeba core by introducing a genetic algorithm (GA). A method that incorporates a GA for dynamic adjustment of the parameters to the base algorithm is called a "hybrid-GA" or "memetic algorithm[S1-S3]." This approach has been reported to be effective for Ising machines[S4] and is expected to be beneficial for our purpose as well, although there is an overhead for the communication between the electronic amoeba and a conventional computer.

Figure S4 shows a flowchart of the electronic amoeba-GA hybrid approach. The system has many electronic amoebae and assigns random resistance values to all or some of the units. The randomly assigned resistance values are regarded as individuals in the GA. Then, the electronic amoebae start the solution search simultaneously. When we obtain several legal solutions, their qualities are evaluated. The combination of the resistance values in the amoeba core that finds a higher-quality solution is selected to take over to the next generation. To promote the diversity in each generation, we apply the "crossover" to swap the resistance value between the selected combinations and introduce the "mutation" to change the values randomly. The combinations of the updated resistance values are fed into the units of the electronic amoebae, and the parallel solution search start again. Repeating these operations, it is expected that the qualities of the solutions found by the electronic amoebae would be improved collectively.

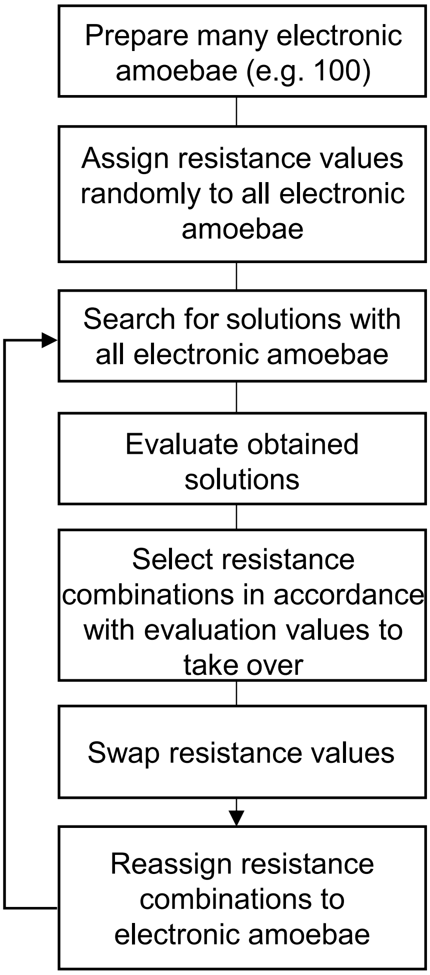

Figure S4 Flowchart of Amoeba-GA hybrid approach.

3.2 Fluctuation-imposing approach

To improve the solution quality, the mathematical model of the amoeba-based computing system, AmoebaTSP[38], introduced stochastic noise to fluctuate the state variable temporally, which resulted in effective trial-and-error dynamics for exploring a wider state space. In the case of the electronic amoeba, if the time scale of the dynamics of the amoeba core could be set to be larger than that of the temporal fluctuation, similar trial-and-error dynamics would be feasible and effective. We can verify this hypothesis by numerical simulation. We formulate the solution-searching dynamics of the electronic amoeba as the following time evolution equations:

$$X_{Vk}(t+1) = \begin{cases} X_{Vk}(t) + \Delta_{IN} \;/\; Light_{OFF}(t+1) & (\text{if } L_{Vk}(t+1) > T) \\ X_{Vk}(t) - X_{Vk}(t) \;/\; \Delta_{OUT} & (\text{if } L_{Vk}(t+1) \leq T) \end{cases}, \quad \text{(S3)}$$

$$L_{Vk}(t+1) = 3\sigma_{\gamma_3,\eta_3}\left(\sum_{Ul}\left(-W_{Vk,Ul}\right) \cdot 3\sigma_{\gamma_4,\eta_4}(X_{Ul}(t) + \delta_{Ul})\right), \quad \text{(S4)}$$

where $Light_{OFF}(t+1)$ is the number of the units without applying the bounceback signals (i.e., an analogy of the number of amoeba branches at which the lights are turned off) at an iteration time step $t+1$, Δ_{IN} and Δ_{OUT} correspond to the expanding and shrinking rates (velocities) of the branch, respectively, and δ_{Ul} is random noise to represent stochastic fluctuation. The numerical simulation is conducted by iterating the calculations of equations (S3) and (S4) successively.

Examples of the time series obtained from the simulation for solving a 10-city TSP instance is shown in Figure S5, where a representative dynamics of state variable $X_{1,0}$ and the outputs of the sigmoid function $\sigma_{15,1.5}\left(X_{1,0} + \delta_{1,0}\right)$ are plotted for different noise amplitudes (real intervals) of $\delta_{1,0}$. As shown in Figs. S5(a)–S5(c), each profile of $X_{1,0}$ first decreases rapidly, reaches a plateau and then decreases further. The duration of the plateau increases with the increase of the noise amplitude. As the state variable $X_{1,0}$ decreases, the noisy output of the sigmoid function $\sigma_{15,1.5}$ decreases as well, enabling the former to converge to zero stably at the end of the solution search even though the latter is still imposed.

Figures S6(a) and S6(b) show the solution quality and the number of iteration steps required for finding a legal solution, respectively. The solution quality increases as the noise amplitude of δ_{Vk} becomes larger. Particularly, the solution quality improves rapidly when $\delta_{Vk} > 1.2$, but finding such a high-quality solution requires longer time steps, indicating the trade-off between quality and time. The minimum route length found by the simulation at a large δ_{Vk} of 1.27 was 845.1, which was close to the minimum route length of 841.09 obtained by the brute-force search.

Figure S7(a) shows a possible circuit design of each unit in the amoeba core whose threshold can be modulated by the external noise. The output voltage X_{Vk} is equal to $X_{Vk} =$

$(1 + R_3/R_2)V_C - (R_3/R_2)(V_T + \delta_{Vk})$ under $V_{SS} \leq X_{Vk} \leq V_{DD}$, where V_T is the threshold voltage and δ_{Vk} is the fluctuation. Figures S7(e)–S7(g) show the output waveforms of the sigmoid function in the circuit (Fig. S7(a)) with external noise indicated by Figs. S7(b)–S7(d), respectively. The sigmoid curve fluctuates largely when large noise is imposed. We stress here that when averaging X_{Vk}, the dynamic range of the nonlinear output is extended by adding large noise, which is sometimes explained in the context of stochastic resonance[S5,S6]. We consider that the fluctuation-imposing approach presented above enables the electronic amoeba to explore a larger variety of candidate solutions, which has to be verified experimentally in the future.

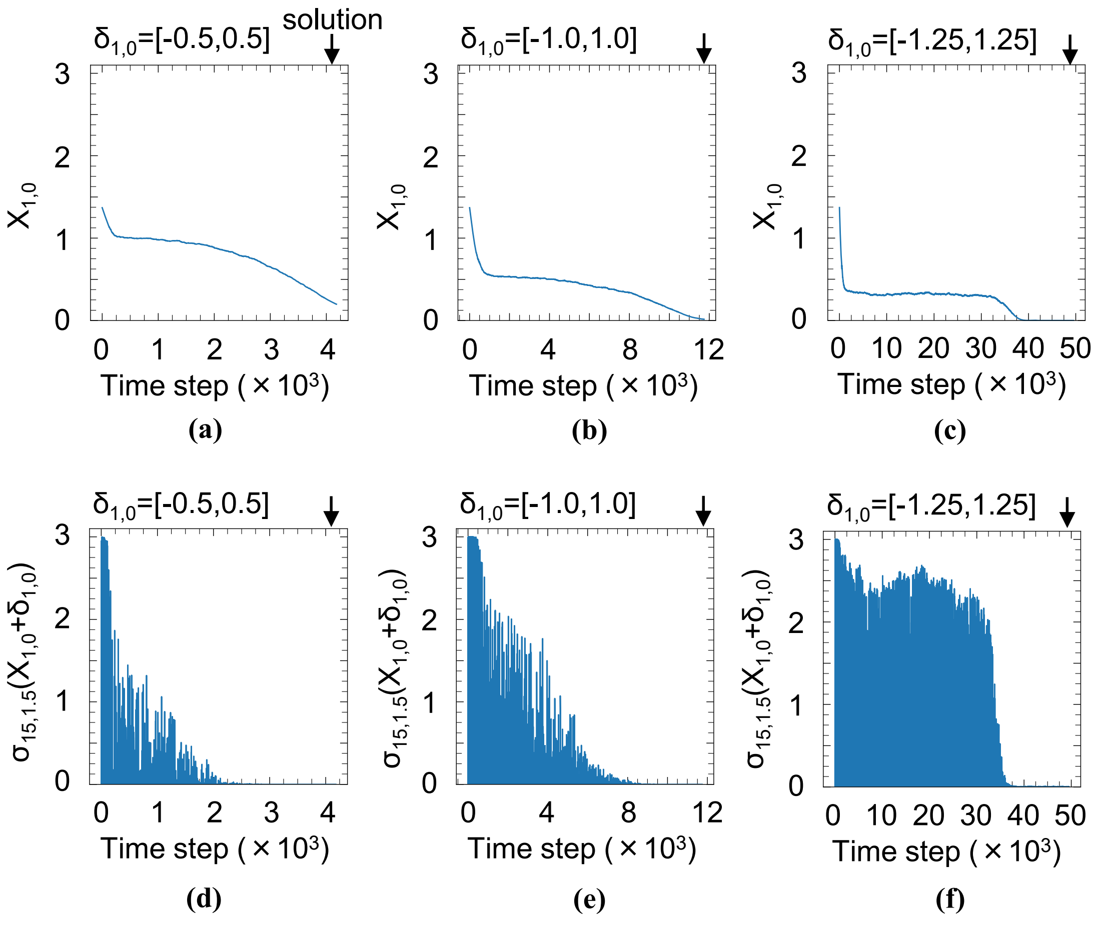

Figure S5 Examples of output waveforms when solving the 10-city TSP instance at $\Delta_{IN} = 0.015$, $\Delta_{OUT} = 650$, $\gamma_3 = 1000$, $\eta_3 = 1.5$, $\gamma_4 = 15$, $\eta_4 = 1.5$ and $T = 0.5$. Initial values of X_{Vk} are all 1.37. (a)–(c) and (d)–(f) are the time evolution of $X_{1,0}$ and $\sigma_{15,1.5}(X_{1,0} + \delta_{1,0})$,

respectively. The amplitude of fluctuation for (a) and (d) is $\delta_{1,0} = [-0.5,0.5]$, that for (b) and (e) is $\delta_{1,0} = [-1.0,1.0]$, and that for (c) and (f) is $\delta_{1,0} = [-1.25,1.25]$.

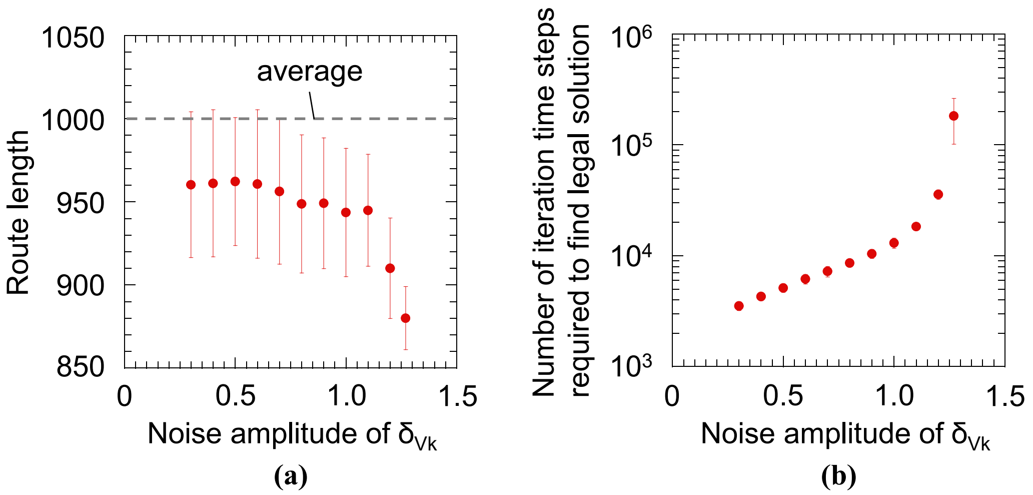

Figure S6 Solution-searching characteristics of the electronic amoeba with fluctuation evaluated by simulation. (a) Obtained route length for solving the 10-city TSP instance. Error bar is derived from 100 trials. (b) Number of steps to reach the solution.

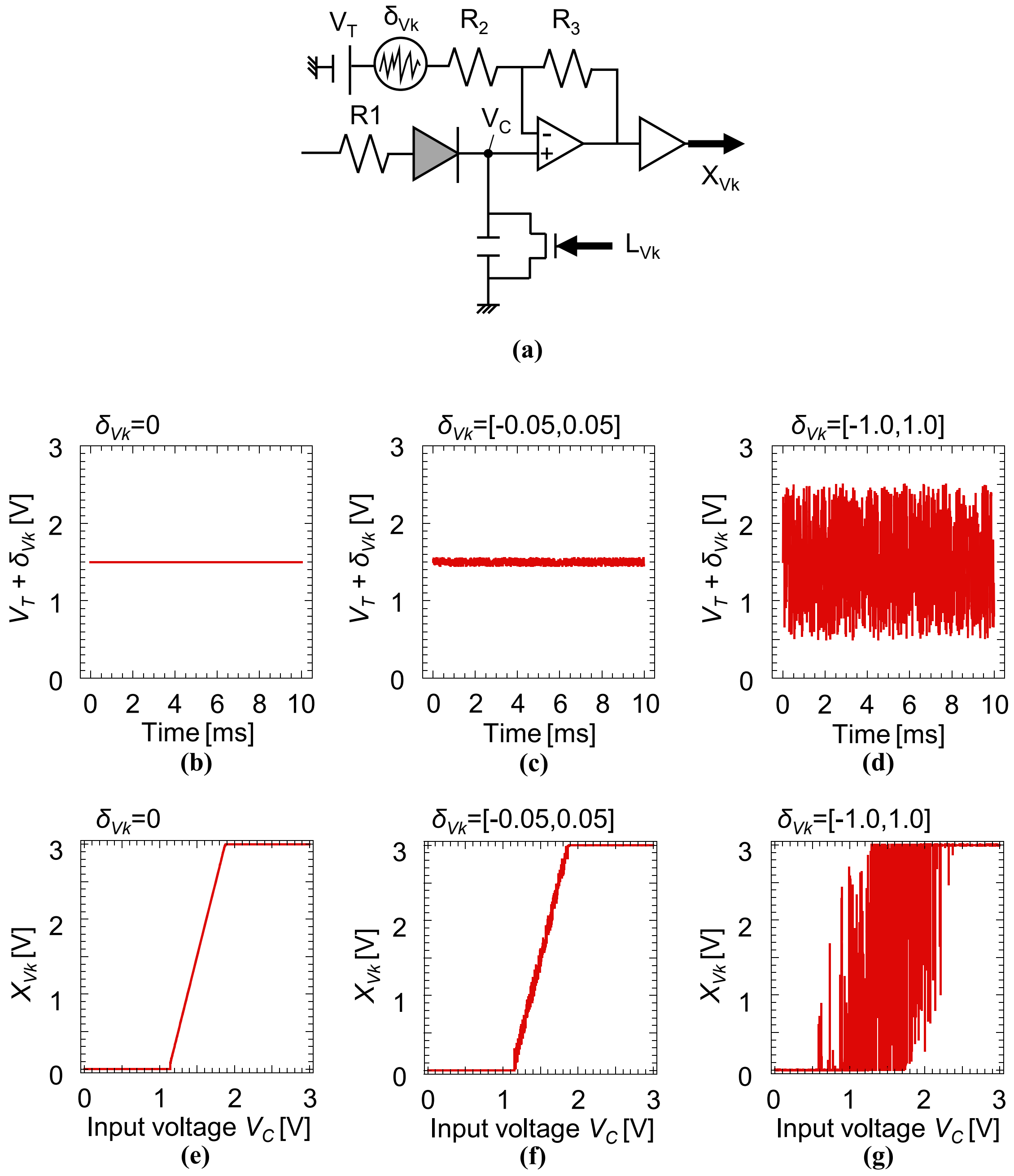

Figure S7 (a) Unit circuit having a sigmoid function whose threshold can be fluctuated by external noise. (b)–(d) and (e)–(g) $V_T + \delta_{Vk}$ and X_{Vk} , respectively. The amplitude of fluctuation δ_{Vk} for (b) and (e) is 0, that for (c) and (f) is $[-0.05,0.05]$, and that for (d) and (g) is $[-1.0,1.0]$.

4. Solution-searching performance of the Ising models

The Ising model searches for a spin assignment that minimizes the Hamiltonian of energy. The Hamiltonian of the Ising model is defined with a cost function and penalty term[S7] as follows:

$$H = H_{cost} + \lambda H_{penalty}\,, \tag{S5}$$

where H_{cost} and $H_{penalty}$ are the cost function and penalty term, respectively, and λ is a coefficient to tune the weight of the penalty term. The cost function is responsible for the quality of a solution and takes a lower value when the length of a route selected is shorter. The penalty term is to ensure the legality of the solution and is designed to increase the energy when the system goes into an illegal state violating the TSP constraints. Therefore, it is a very important to optimize λ to obtain a high-quality legal solution[29]. When λ is relatively large, the Ising model tends to find a legal solution that is close to that obtained by random sampling due to the minimal impact of the cost function on the Hamiltonian. On the other hand, when λ is too small, the system tends to reach an illegal state, which represents an infeasible route. Hence, to determine the optimal λ value, a user of the Ising model has to perform many solution-searching runs until it succeeds in finding a legal solution. For this reason, D-Wave's Ising machine[17] requires to perform the post-processing for repairing the converged spin alignment to satisfy the constraints and for improving the solution quality[2,S8]. The similar treatments are also necessary for the Hopfield's neural network to obtain a legal solution. In the case of the electronic amoeba, however, these costly pre-processing and post-processing are not necessary.

We evaluate the solution-searching performance of the Ising model-based algorithms for comparison with that of the electronic amoeba. The Ising model for solving the TSP is formulated on the basis of quadratic unconstrained binary optimization (QUBO) given as follows[S9]:

$$E = \alpha \sum_{v} \left(1 - \sum_{j} x_{v,j}\right)^2 + \alpha \sum_{j} \left(1 - \sum_{v} x_{v,j}\right)^2 + \sum_{u,v} W_{uv} \sum_{j} x_{u,j} x_{v,j+1}\,, \tag{S6}$$

where $x_{v,j} = 1$ denotes that a salesman visits city v at the jth order, W_{uv} is the distance between cities u and v and α is a parameter to tune the weight of the first two penalty terms. An assignment of the variables at the minimum value of E represents an optimal solution of the TSP. Equation (S6) can be converted to the Hamiltonian of the Ising model as $x_{v,j} = (\sigma_{v,j} + 1)/2$, $\sigma_{v,j} \in \{-1,1\}$, where $\sigma_{v,j}$ represents the spin at lattice site (v, j). Figures S8(a) and S8(b) show flowcharts of the two algorithms for exploring a variable assignment that minimizes E in equation (S6), which are the Ising-model-based algorithms without and with simulated annealing (SA), respectively (referred to as "Ising model" and "Ising model SA").

Figure S9 shows the simulation results of the Ising model. For each N, when α of the Ising model was set at $\alpha = 100$ that resulted in finding a legal solution whose quality was comparable to that found by the electronic amoeba (Fig. S9(a)), the rate of simulation trials in which the Ising model found a legal solution out of 100 trials was only around 20% (Fig. S9(b)). Note that the Ising model succeeds in converging at a legal solution by setting α large enough, such as $0 < \max(W_{uv}) < \alpha$. For each N, when α was set at $\alpha = \max(W_{uv}) + 1$ that ensured to find a legal solutions with a success rate of 100% (Fig. S9(d)), the quality of a legal solution found by the Ising model degraded and could not be comparable to that found by the electronic amoeba (Fig. S9(c)). On the other hand, a success rate of the electronic amoeba to find a legal solution was always 100%.

Figure S10 shows results of the Ising model SA. When $\alpha = 100$, the solution quality of the Ising model SA was further improved by reducing the temperature gradient k and became higher than that of the electronic amoeba (Fig. S10(a)). However, with the increase in the number of cities N, the success rate of finding a legal solution decreased as shown in Fig. S10(b). When $\alpha = \max(W_{uv}) + 1$, although the Ising model SA found a legal solution with a success rate of 100% (Fig. S10(d)), the solution quality degraded considerably to a level that was even worse than that of the electronic amoeba (Fig. S10(c)). These results confirm that the electronic

amoeba is more useful than the Ising machines for practical applications that require to obtain a legal solution reliably and swiftly.

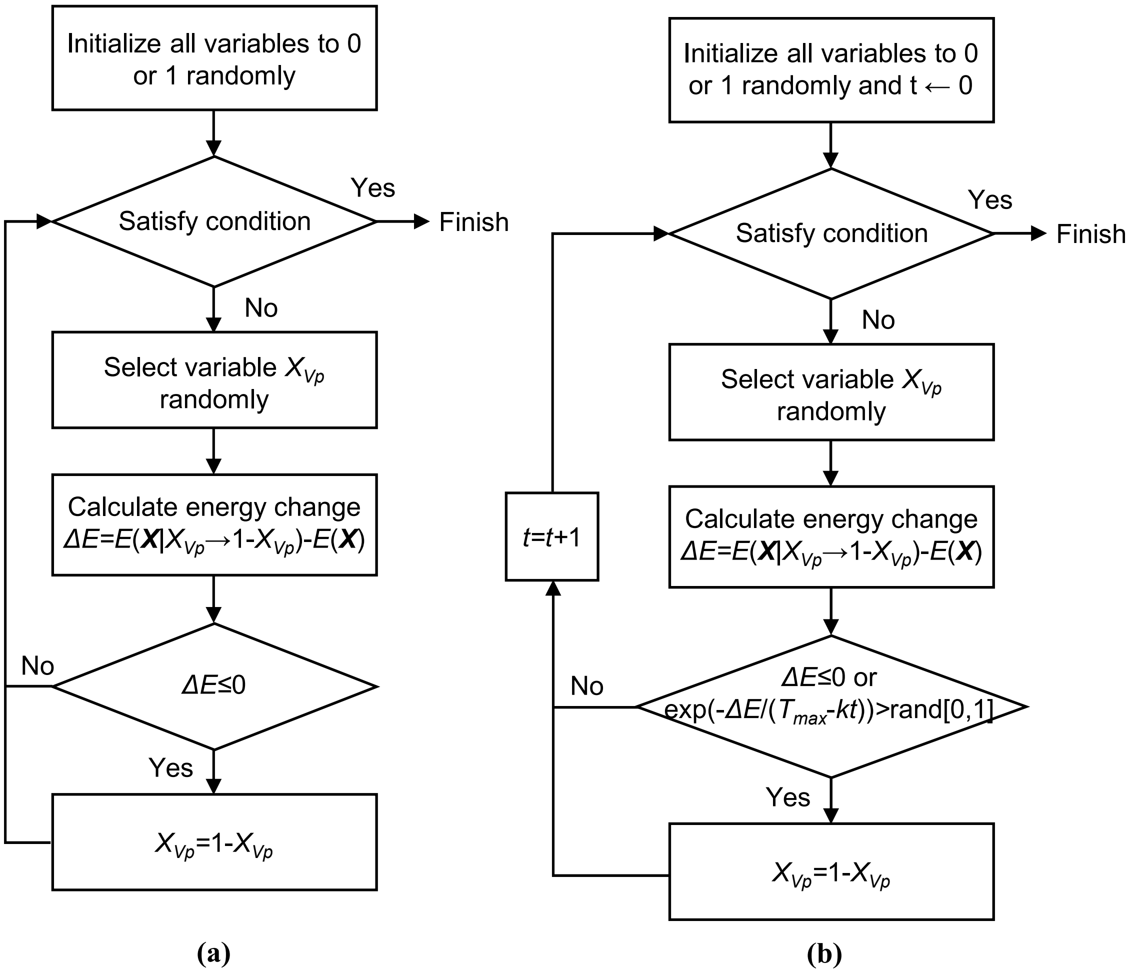

Figure S8 Flowcharts of Ising model-based algorithms for TSP (a) without SA and (b) with SA.

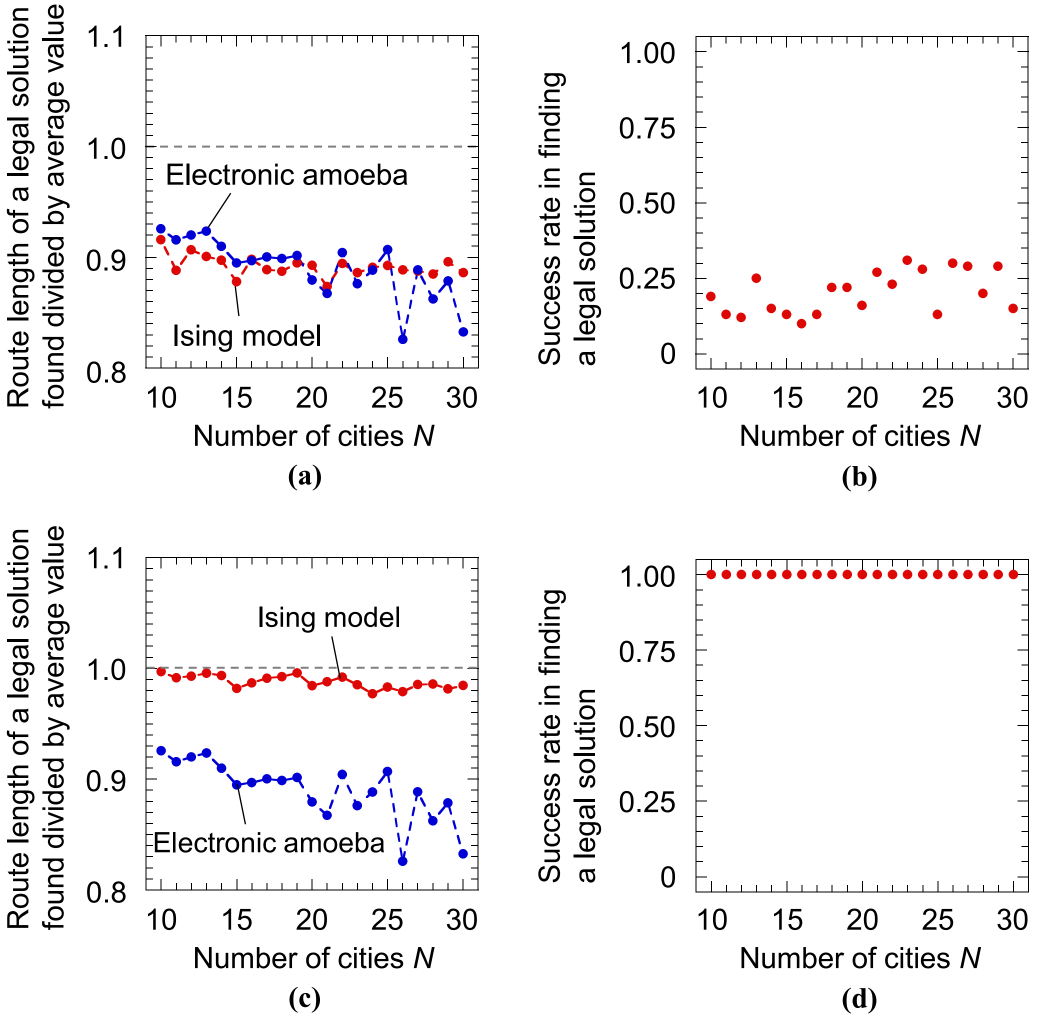

Figure S9 Simulation results of the Ising model for the TSP. (a) Solution quality and (b) success rate of finding a legal solution when $\alpha = 100$, where the iterations continued until the quality of the solution found by the Ising model became comparable to that found by the electronic amoeba. The solution quality is an average value of the solutions found. (c) Solution quality and (d) success rate of finding a legal solution when $\alpha = \max(W_{uv}) + 1$. We performed 100 simulated trials for each instance.

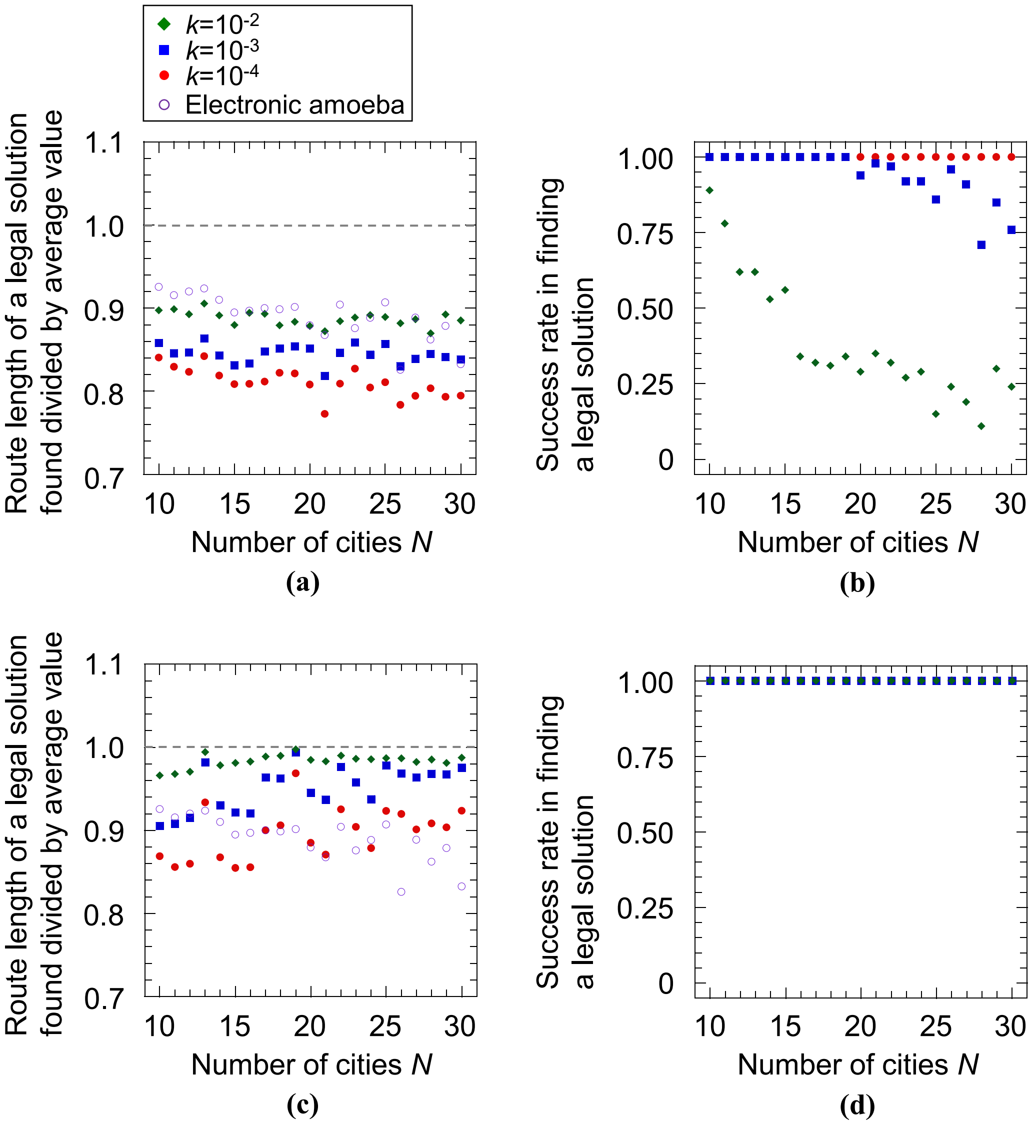

Figure S10 Simulation results of the Ising model SA for the TSP. (a) Solution quality and (b) success rate of finding a legal solution when $\alpha = 100$ and $T_{max} = 30$. (c) Solution quality and (d) success rate of finding a legal solution when $\alpha = \max(W_{uv}) + 1$ and $T_{max} = 30$. Filled green rhombuses, blue squares, red circles and empty purple circles indicate results of $k = 10^{-2}$, 10^{-3} , 10^{-4}, and the electronic amoeba, respectively. The number of iterations was set to $T_{max}/k + 10000$. We performed 100 simulated trials for each instance.

References

S1. Watanabe, Y., Mizuguchi, N. & Fujii, Y. Solving optimization problems by using a

Hopfield neural network and genetic algorithm combination. *Syst. Comput. Japan* **29**, 68–74 (1998).

S2. Salcedo-Sanz, S. & Yao, X. A hybrid Hopfield network-genetic algorithm approach for the terminal assignment problem. *IEEE Trans. Syst. Man, Cybern. Part B (Cybern.)* **34**, 2343–2353 (2004).

S3. Matayoshi, M., Nakamura, M. & Miyagi, H. A Genetic Algorithm with the Improved 2-opt Method. *IEEE International Conference on Systems, Man and Cybernetics* **4** 3652–3658 (2004).

S4. King, J. *et al*. Quantum-Assisted Genetic Algorithm. Preprint at http://arxiv.org/abs/1907.00707 (2019).

S5. Gammaitoni, L. Stochastic Resonance and the Dithering Effect in Threshold Physical Systems. *Phys. Rev. E* **52**, 4691–4698 (1995).

S6. Tadokoro, Y., Kasai, S., Ichiki, A. & Tanaka, H. Design Framework of Image Sensor System Based on Dynamic Range Extension by Adding Noise for Saturated Conditions. *IEEE Trans. Systems, Man, and Cybernetics: Systems* **48**, 1121–1128 (2016).

S7. Tanahashi, K., Takayanagi, S., Motohashi, T. & Tanaka, S. Application of Ising machines and a software development for Ising machines. *J. Phys. Soc. Japan* **88**, 061010 (2019).

S8. D-Wave Systems: Postprocessing Methods on D-Wave Systems (2018).

S9. Lucas A. Ising Formulations of Many NP Problems. *Front. Phys.* **2**, 1-15 (2014).
